# Humor Affects Fairness Considerations in the Gain and Loss Contexts

**DOI:** 10.3389/fpsyg.2018.02679

**Published:** 2019-01-07

**Authors:** Zhong Yang, Di Fu, Yue Qi, Ya Zheng, Qi Li, Xun Liu

**Affiliations:** ^1^CAS Key Laboratory of Behavioral Science, Institute of Psychology, Chinese Academy of Sciences, Beijing, China; ^2^Department of Psychology, University of the Chinese Academy of Sciences, Beijing, China; ^3^Department of Psychology, Dalian Medical University, Dalian, China

**Keywords:** humor, emotion, unfair, gain, loss, Ultimatum Game

## Abstract

Human decision-making behaviors in social contexts are largely driven by fairness considerations. The dual-process model suggests that in addition to cognitive processes, emotion contributes to economic decision-making. Although humor, as an effective emotional regulation strategy to induce positive emotion, may influence an individual’s emotional state and decision-making behavior, previous studies have not examined how humor modulates fairness-related responses in the gain and loss contexts simultaneously. This study uses the Ultimatum Game (UG) in gain and loss contexts to explore this issue. The results show, in the gain context, viewing humorous pictures compared to humorless pictures increased acceptance rates and this effect was moderated by the offer size. However, we did not find the same effect in the loss context. These findings indicate that humor’s affection for fairness considerations may depend on the context and provide insight into the finite power of humor in human sociality, cooperation and norm compliance.

## Introduction

Since ancient times, human behaviors in social contexts have been largely driven by fairness considerations. For example, the Bible says, “To do right and justice is more acceptable to the Lord than sacrifice,” and the Confucian Analects say, “Inequality rather than want is the cause of trouble.” Decades of work in economics have shown that fairness can prompt interpersonal cooperation and the development of social economics, whereas unfairness may decrease interpersonal trust during social interactions and forestall cooperation (Brosnan and de Waal, 2014; Chin and Culotta, 2014; Piketty and Saez, 2014). As one of the most fundamental social norms, fairness plays a significant role in the advancement of human civilization (Zhou et al., 2014).

An increasing number of studies have used the Ultimatum Game (UG) to illustrate the influence of fairness on decision-making (Güth et al., 1982). In this game, two players split a sum of money. One player assumes the role of the proposer, and the other is the responder. The proposer can decide how to split the money between the two players, and the responder can decide to accept or reject the proposal. If the offer is accepted, then the money is split as proposed; however, if the responder rejects the offer, then both players receive nothing. Previous UG studies have found that unfair offers, especially offers below 20% of the total, are rejected by many responders (Henrich et al., 2001; Sanfey et al., 2003). These findings cannot be captured by standard economic models, which predict that responders should make the rational decision to maximize their benefits and accept any monetary amount.

The dual-process model attempts to explain why responders reject unfair offers in the UG. It posits that in addition to the existence of a rational system, there is an emotional system that influences the responder’s decision. When an offer is low, it is often perceived as unfair and is followed by a negative emotional reaction, which leads people to give up financial gain to punish their partner (Sanfey and Chang, 2008). This model is supported by previous research. On the one hand, the player’s emotional state may be the reason for the rejection of unfair offers in the UG. For example, using clips (Harle and Sanfey, 2007; Andrade and Ariely, 2009), emotional pictures (Moretti and Di Pellegrino, 2010), imaging (van den Bos, 2003), trait-valenced words (Maltese et al., 2016), and unpleasant smells (Bonini et al., 2011) to arouse emotion, previous studies have shown that inducing positive emotion increases the acceptance rates of unfair offers, whereas inducing negative emotion decreases the acceptance rates of unfair offers. Furthermore, correlation analysis has found that negative emotion is significantly correlated with unfairness ratings (Pillutla and Murnighan, 1996). On the other hand, emotion regulation strategies may modulate the rejection of unfair offers in the UG. Using two different regulation strategies in the UG (expressive suppression and emotional reappraisal), research has found that compared with expressive suppression, emotional reappraisal is a more powerful downregulation strategy to influence the reaction to inequity (van’t Wout et al., 2010). Furthermore, decision acceptance rates are altered during reappraisal, with higher acceptance rates of unfair offers while downregulating (reappraising the proposer’s intentions as less negative) and lower acceptance rates of unfair offers while upregulating (reappraising the proposer’s intentions as more negative), both relative to the baseline condition (Grecucci et al., 2012). Moreover, research has found that the positive or negative anticipated emotions may influence the fairness of subsequent behavior in the UG (van der Schalk et al., 2012). A common finding among these studies is that increasing positive emotion or decreasing negative emotion may increase the acceptance rates of unfair offers in the UG in the gain context. However, more attention should have been paid in loss contexts. In comparison with gain contexts, a proposer and a responder split a sum of debt in loss contexts. The proposer can give an offer, and the responder can accept or reject the proposal. If the offer is accepted, then the debt is split as proposed; however, if the responder rejects the offer, then both players have to pay the total amount of the debt. According to prospect theory, losses loom larger than gains, and people prefer to avoid losses rather than to acquire equivalent gains (Güth et al., 1982; Buchan et al., 2005; Zhou and Wu, 2011). Individuals may perceive stronger unfairness and to reject more unequal offers in loss context (Guo et al., 2013; Sarlo et al., 2013; Tomasino et al., 2013). To our knowledge, no research has explored the way that positive emotion affects responses in the UG in the loss context.

As an effective emotional regulation strategy to induce positive emotion, humor may influence individuals’ emotional state and decision-making behavior. First, humor may increase positive emotion and reduce negative emotion. Studies have found that humor is an effective means of up- and downregulating negative and positive emotions (Giuliani et al., 2008; Samson and Gross, 2012) and that humorous stimuli attenuate negative emotions to a greater extent than do equally positive non-humorous stimuli (Strick et al., 2009). Second, humor increases participants’ tendency to favor utilitarian solutions to social decision-making. Some research has reported that humorous videos increase positive affect, which makes people more likely to choose a utilitarian solution to a moral dilemma (Valdesolo and DeSteno, 2006). Other research has found that these results stem from the specific mirth properties of humor rather than general positive affect (Strohminger et al., 2011). Furthermore, neuroimaging and EEG studies have shown that a network of subcortical regions are associated with humor, including the reward-related nucleus accumbens area and the emotional area of the amygdala (Mobbs et al., 2003; Vrticka et al., 2013; Mensen et al., 2014). Although humor can modulate individuals’ emotional state and can cause decision-making to present a utility orientation, it remains unclear whether humor can cause responders to make utilitarian decisions and accept unfair offers.

Human fairness decision-making behavior differs in gain and loss contexts. Although many studies have shown that equivalent value in decision-making is greater in loss contexts than that in gain contexts (Li et al., 2011, 2016a,b), few studies have investigated this issue in strategic situations. One study identified a difference in the UG for gains and losses and found that proposers would propose higher offers and the responders demand higher in loss context than those in gain context, suggesting that unfairness loom larger than unfairness in gain (Buchan et al., 2005). Another study found that unfair losses were perceived as more unfair than unfair gains in subjective ratings, leading to lower acceptance rates in the loss context than those in the gain context (Zhou and Wu, 2011). An fMRI study revealed consistent results that participants may experience more unfairness in the UG and are more inclined to punish social norm violations in the loss context than in the gain context, thereby inducing more fairness-related neural activities when rejecting (vs. accepting) unfair losses rather than unfair gains (Guo et al., 2013). Therefore, whether humor has the same influence on fairness decision-making in gain and loss contexts is yet to be determined.

To resolve the above issues, this study investigated whether humor affects individuals’ rejection rates of the UG in a gain context and in a loss context by using humorous and humorless pictures as materials to induce positive emotion. We hypothesized that compared with humorless pictures, humorous pictures would arouse individuals’ positive emotion, which leaded the participants to accept more unfair offers in the gain context. However, loss looms larger than gain, and people prefer to avoid losses rather than to acquire equivalent gains according to previous studies (Güth et al., 1982; Buchan et al., 2005). Therefore, we predicted that positive emotion induced by humorous stimuli could not sufficiently modulate fairness decision-making in the loss context as in the gain context did.

## Materials and Methods

### Participants

Non-psychology and economic majors at a university in Beijing were recruited as participants through the university’s Bulletin Board System. None of the participants reported that they had previously taken part in the similar experiments. Fifty-one (12 males, 22.84 ± 2.41 years old) and forty-eight (12 males, 21.85 ± 2.19 years old) university students participated in gain context and loss context, respectively. We estimated an optimal sample size by using G^∗^Power 3.1 for α = 0.05, a power (1 – β) = 0.9, and a medium effect size = 0.25 (Cohen, 1969). Therefore, 99 participants in our experiments were sufficient to identify the significant differences. All participants reported a lack of neurological or psychiatric history. Each participant voluntarily enrolled and signed an informed consent statement prior to the experiment. The procedure was approved by the Institutional Review Board of the Institute of Psychology, Chinese Academy of Sciences.

### Stimuli

Humorous and humorless pictures were selected from a popular amusement comic website^1^. The content of the comics from this website mainly involve embarrassing but funny moments in daily life, so the comics are easily understood and resonate with viewers. Thirty-two participants (9 males, 23.50 ± 2.90 years old) were recruited to measure the amusement level of 209 comic pictures using a scale from 1 (very humorless) to 10 (very humorous). Pictures were ranked in the order of the mean scores from highest to lowest, with the top 23% (M ± SD = 6.01 ± 0.33, *n* = 48) selected as humorous stimuli and the last 23% (M ± SD = 3.76 ± 0.39, *n* = 48) selected as humorless stimuli. The paired *t*-test showed that humorous stimuli were significantly more amusing than humorless stimuli, *t* = 69.30, *p* < .001.

### Procedure

Before the participants enrolled in the study, they were asked to submit a self-introduction, including their major, upbringing, education, personality, and hobbies. They were told that their self-introduction would be anonymously supplied to some proposers who would split a sum of money with them according to the content of their self-introduction. Furthermore, the participants were told that on each trial of the formal experiment, the offers were given by a different proposer and all these offers had been entered into a computer in advance for display.

**FIGURE 1:**
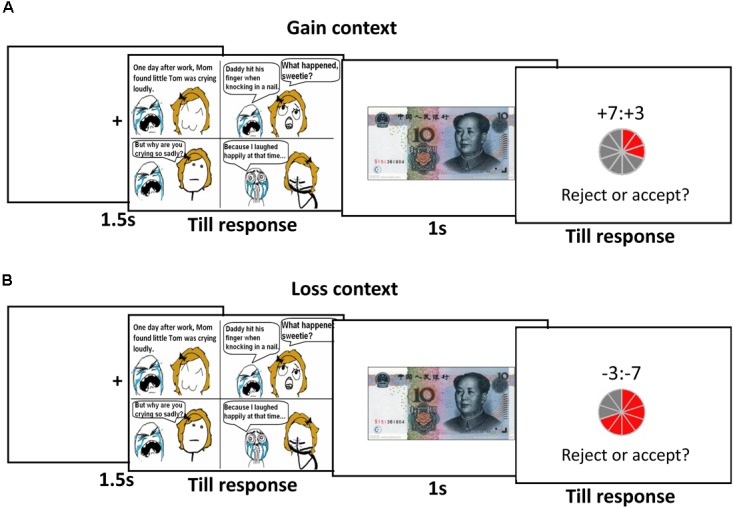
Experimental procedure. **(A)** gain context and **(B)** loss context.

On the day of the experiment, the participants were asked to evaluate their mood on a 7-point scale ranging from 1 (extremely unhappy) to 7 (extremely happy). Firstly, the participants viewed a comic picture; some of the comics were humorous, whereas others were humorless (see Figure 1). The participants were told that they could laugh loudly and freely while viewing the comic and that no one could hear them. Then, the offers proposers proposed which were involved in splitting a gain or loss of ¥10 were presented on the screens. A pie divided into 10 equal parts, with each part representing ¥1, represented the ¥10, with the red part indicating the participant and the gray part indicating the proposer. When the offer was given, as responders, the participants were required to press the F key with their left index finger for an acceptance decision and to press the J key with their right index finger for a rejection decision. In the gain context, accepting the offer led to the suggested division of the gain, whereas rejection resulted in both players receiving nothing. In the loss context, accepting the offer led to the suggested division of the loss, whereas rejection resulted in both players incurring the entire loss. All participants were told that none of the proposers would receive the money until all participants finished the experiments. Their decisions determined the payment between themselves (responders) and the proposers. After completing the study, the participants were given payment for their participation (¥ 40) plus or minus the amount of money they obtained or lost from a random selection of 10% of the trials of the game in gain context and loss context, respectively. Each participant only engaged in one context. For each participant, the context and the response buttons were randomly determined.

Each context consisted of 96 trials divided evenly into four runs, two runs with humorous pictures and two runs with humorless pictures. All trials were presented randomly, and no more than 3 consecutive trials had the same humor type or offer type. The offer types were 5:5, 6:4, 7:3, 8:2 and 9:1 with increased unfairness levels in the gain and the loss context. However, the first number represented the payoff for the proposer, and the second number represented the payoff for the responder in the gain context. In contrast, the first number represented the amount of loss for the responder, and the second number represented the amount of loss for the proposer in the loss context. Moreover, 16 trials of hyper-fair levels were used only to enhance the sense of reality. At the end of each run, the participants were asked to rate their mood on a 7-point scale ranging from 1 (very unhappy) to 7 (very happy).

At the beginning of each trial, a fixation cross was presented for 1.5 s, and then the participants observed a humorous picture or a humorless picture until they understood the picture and pressed the “space” key. Next, a ¥10 picture was presented for 1 s. The offer was displayed, and the participants were required to make an acceptance or a rejection decision by pressing a button. After the experiments, all participants were asked to report whether they suspected the offers were not come from real proposers and nobody suspected the offers were not genuine. Then, they were told that no real proposers existed and that all of the participants had been given the same offers regardless of the content of their self-introductions.

## Results

### Emotion Manipulation

**FIGURE 2:**
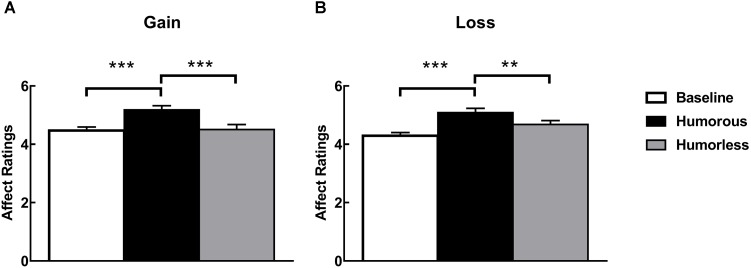
Self-reported affect ratings in **(A)**^∗∗^” specified in Figure 2. a gain context and **(B)** a loss context. The asterisk (^∗∗^) and (^∗∗∗^) represents the significant difference of the *post hoc* Bonferroni tests at the *p* < 0.01 level and *p* < 0.001, respectively.

For the affect ratings, there are three emotional conditions: the baseline emotion before the experiment and the emotional states induced by watching humorous or humorless pictures. Thus, a 3 (emotional conditions: baseline vs. humorous vs. humorless) ^∗^ 2 (context: gain vs. loss) mixed design ANOVA was conducted. The results revealed significant main effects of emotional states [*F*(2,194) = 22.43, *p* < 0.001]. The *post hoc* analysis showed that positive affect ratings in the humorous condition were significantly greater than those in the baseline and humorless conditions (*ps* < 0.001), but no significant difference was observed between the baseline and humorless conditions (*ps* > 0.05). No other main effects and interactions were significant (see Figure 2).

### Acceptance Rates in UG Tasks

For the acceptance rates, a 2 (humor type: humorous vs. humorless) ^∗^ 5 (offer type: 5:5 vs. 6:4 vs. 7:3 vs. 8:2 vs. 9:1) ^∗^ 2 (context: gain vs. loss) mixed design ANOVA was conducted. The main effect of the humor type was significant, *F*(1,97) = 9.87, *p* < 0.01, η^2^ = 0.09, indicating higher acceptance rates after viewing humorous pictures (M ± SE = 64.32% ± 2.28) than after viewing humorless pictures (M ± SE = 61.74% ± 2.27). The main effect of offer type was significant, *F*(4,388) = 170.36, *p* < 0.001, η^2^ = 0.64. The *post hoc* analysis indicated decreased acceptance rates with the unfairness levels, *ps* < 0.001. The interaction between the humor type and the context was significant, *F*(1,97) = 13.77, *p* < 0.001, η^2^ = 0.12. The simple effect analysis indicated that it was higher after participants viewed humorous pictures than after they viewed humorless pictures in gain context (67.55% ± 3.18 vs. 61.91% ± 3.16, *ps* < 0.001), but this effect of humor disappeared in loss context (61.09% ± 3.28 vs. 61.56% ± 3.25, *ps* > 0.05). No other main effects and interactions were significant.

**FIGURE 3:**
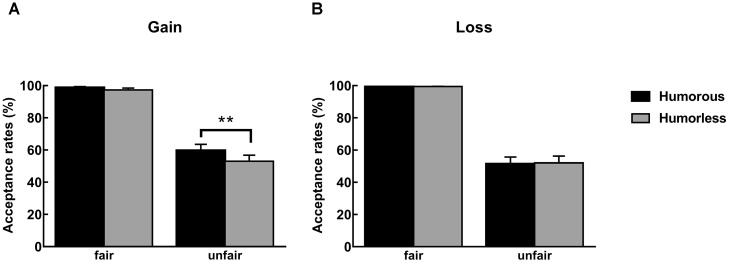
The^∗^” specified in Figure 3. acceptance rates were plotted as a function of unfairness level in **(A)** a gain context and **(B)** a loss context. The asterisk (^∗∗^) represents the significant difference of the *post hoc* Bonferroni tests at the *p* < 0.01 level.

Moreover, we regard the 5:5 as fair offers and the others as unfair offers. Thus, a 2 (humor type: humorous vs. humorless) ^∗^ 2 (offer type: fair vs. unfair) ^∗^ 2 (context: gain vs. loss) mixed design ANOVA was conducted. The main effect of humor type was significant, *F*(1,97) = 7.33, *p* < 0.01, η^2^ = 0.07, indicating higher acceptance rates after viewing humorous pictures (M ± SE = 77.37% ± 1.46) than after viewing humorless pictures (M ± SE = 75.48% ± 1.47), *p* < 0.01. The main effect of the offer type was significant, *F*(1,97) = 261.33, *p* < 0.001, η^2^ = 0.73. The *post hoc* analysis indicated it was higher for fair offer (M ± SE = 98.76% ± 0.36) than unfair offer (M ± SE = 54.10% ± 2.78). The interaction between the humor type, the context and the offer type was significant, *F*(1,97) = 9.10, *p* < 0.01, η^2^ = 0.09. The simple effect analysis indicated that the participants accept more unfair offers after they viewed humorous pictures than after they viewed humorless pictures in gain context (59.74% ± 3.95 vs. 53.06% ± 3.92, *ps* < 0.001), but this effect disappeared in loss context and for fair offers, *ps* > 0.05 (see Figure 3).

## Discussion

In this study, we investigated whether positive emotion induced by humor could affect fairness-related decision-making in gain and loss contexts. Consistent with prior findings, most individuals rejected unfair offers even at personal cost. Rejection rates were a function of the fairness of the offer; that is, people would be more likely to reject the offer if the offer was more unfair in both the gain and loss contexts (Zhou and Wu, 2011). More importantly, the present results extend prior research. To the best of our knowledge, this is the first study to reveal the moderating effect of humor on fairness considerations in the gain and loss contexts simultaneously. Specifically, increased acceptance rates occurred after viewing humorous pictures compared to humorless pictures in the gain context, and this effect was moderated by the type of offer. However, we did not find a significant effect of humor in the loss context. Overall, these findings indicate that humor-related positive emotions may regulate individuals’ emotional state and affect their fairness decision-making behaviors depending on the context.

The present study found that humor increased positive emotion. Previous studies have proposed that humor, as an emotion management tool, increases or decreases the emotional effect of a situation, creates amusement for oneself and others, and generates positive sentiments among individuals facing an external threat (Francis, 1994). In the present study, we observed that, participants reported more positive affect ratings after viewing humorous pictures than after viewing humorless pictures. This finding is consistent with previous research in which participants felt more positive and less negative after making jokes about aversive images (Giuliani et al., 2008; Strick et al., 2009; Samson and Gross, 2012) and experienced less distress when they humorously narrated an aversive video (Waldman et al., 2011).

The present results indicate that compared with viewing humorless pictures, viewing humorous pictures caused the responders to demonstrate higher acceptance rates of unfair offers. It is possible that humor, as a type of emotional regulation strategy, may improve responders’ positive emotion and reduce the negative emotion produced by unfairness offers in the gain context, which leads the responders to forgive and to accept more unfair suggestions. Based on the participants’ reports, low offers that were perceived as unfair were often rejected with an angry reaction (Francis, 1994). Rejecting unfairness has been proposed as a fundamental adaptive mechanism to maintain a social reputation (Nowak et al., 2000). The negative emotions provoked by an unfair offer in the UG may lead people to give up considerable financial gain to punish their partner for fairness norm enforcement (Sanfey and Chang, 2008). Research has found that the positive effect of watching a humorous video at the time of judgment may attenuate the perceived negativity or aversion of a potential moral violation and thus may increase utilitarian responses (Valdesolo and DeSteno, 2006). In this study, humor induced positive emotion, which reduced the negative emotions provoked by unfair treatment and increased the utilitarian response for unfairness behavior.

Alternatively, because of humor’s positivity rather than a general positive emotion associated with non-serious situations, unfair behavior may be more likely to be condoned and accepted. Humor is defined as a psychological state characterized by the positive emotion of amusement, an appraisal that something is funny, and a tendency to laugh (McGraw et al., 2015). Positivity and arousal, which are outcomes of successful attempts at humor, drive sharing behavior (Jonah and Milkman, 2012). Some research has found that increased permissiveness toward deontological violations in moral judgments stem from the specific properties of humor rather than from general positive affect, such as elevation, suggesting that humor may cause people to condone indiscriminate moral behavior (Strohminger et al., 2011). Furthermore, evolutionary theories suggest that humor is triggered by a false alarm (Ramachandran, 1998) and that the laughter aroused by humor is often associated with harmless attacks, such as tickling and roughhousing (Rich, 2001; Gervais and Wilson, 2005). Humor may cause a seemingly important situation to be reinterpreted as unimportant (Rilling and Sanfey, 2011) or as a benign violation (McGraw and Warren, 2010; McGraw et al., 2013). Many studies have shown that attempts at humor, such as jokes, puns, and slapstick, are typically not intended to be taken seriously, and humor often occurs in casual conditions (Gervais and Wilson, 2005; Martin, 2010). Therefore, after viewing the humorous pictures, humorous reactions were usually associated with the appraisal that an unfairness situation was safe, and laughter due to humor as a signal indicated to the responder that the unfairness situation was not intended to be harmful (Ramachandran, 1998; Gervais and Wilson, 2005; McGraw et al., 2012).

Our study did not find an effect of humor on fairness decision-making in the loss context. In the present study, we employed the same materials to induce equally positive emotion in both the gain and loss contexts. Similar to the gain context, in the loss context, participants reported higher positive affect ratings after viewing humorous pictures than after viewing humorless pictures. However, no difference in acceptance rates was found between these two conditions. Some explanations may account for the non-significant effect of humor in the loss context. One possibility is that the intensity of emotion evoked by humor is insufficient to change fairness decision-making behavior in the loss context. Although our current research employed the classic paradigm of the UG in the gain-or-loss context, it was clear that rejection yielded non-equivalent results in different situations. In the gain context, the rejection of unfair distribution meant only that the participant did not receive a reward. However, in the loss context, rejection meant that the participant paid money. Previous studies have found that bargaining in the loss context requires higher demands on the part of responders and higher offers on the part of proposers than bargaining in the gain context, suggesting that unfairness seems greater in the loss context than that in the gain context (Zhou and Wu, 2011; Wu et al., 2014; Yin et al., 2017). Although, as shown in previous studies, the humor involved in making individuals laugh can distract them from their existing judgments and opinions and increase their acceptance of unfair offers, the same intensity of positive emotion generated by humor is not sufficient to change fairness decision-making behavior in the loss context. Another possibility is that fairness-related decision-making behavior in the loss context is more difficult to regulate by humor than in the gain context. The essence of humor is that it weakens the seriousness of the current situation (McGraw et al., 2015) so that people are able to view the situation from a broader, more tolerant perspective. Because potential losses have a greater effect on people’s choices than equivalent gains (Tversky and Kahneman, 1992), the participants who experienced unfairness in the UG would have a stronger desire to punish social norm violations in liability sharing than in gain sharing (Guo et al., 2013). Therefore, when faced with loss-related fairness decision-making, it may be difficult to affect individuals’ ideas and ways of thinking by humor; these individuals are likely to address the situation in a seemingly “stubborn” and serious way. Moreover, studies have found that gains and losses, as two independent contexts that are often unrelated, can be differentially affected by the same experimental manipulation (Li et al., 2016b). Besides the acceptance rates, we may use the indifference point as another sensitive index. We could set more variable offer types to explore the change of indifference points of fairness-related decision making after viewing humorous pictures in the gain and loss context. Further discussion is essential to develop the modified paradigm and test the effect of humor on fairness decision-making in the loss context.

Although there are a couple of empirical works have explored the influence of mood on fairness-related decision-making, our study is distinct relative to these previous studies. Firstly, previous studies usually examined the effect of emotions on fairness decision making in the gain context. However, losses loom larger than gains according to prospect theory, and human fairness decision-making behavior differs in gain and loss contexts (Güth et al., 1982; Buchan et al., 2005; Zhou and Wu, 2011). Therefore, it is necessary to determine whether humor has the same influence on fairness decision-making in gain and loss contexts. Secondly, previous studies usually induced emotions via happy-related movie clips (Güth et al., 1982; Buchan et al., 2005; Zhou and Wu, 2011), while the present study used humorous pictures. As an effective emotional regulation strategy to induce positive emotion, humor could increase humor-related positive emotion (Giuliani et al., 2008; Samson and Gross, 2012) and makes people more likely to choose a utilitarian solution to a moral dilemma (Valdesolo and DeSteno, 2006). Therefore, the influence of humor on fairness decision-making stemmed from the specific mirth properties of humor rather than general positive affect (Strohminger et al., 2011).

To summarize, our findings suggest that humor-related positive emotion affects fairness in the gain context, which supports the dual-process model and cannot be captured by standard economic models. Furthermore, the outcomes of the present research demonstrate that fairness considerations may not be affected by humor in the loss context, which may provide insight into the finite power of humor-related positive emotion in human sociality, cooperation and norm compliance. Our findings provide new information on the regulation of people’s fairness considerations and shed light on diverse human behaviors.

## Data Availability Statement

The datasets generated for this study can be found in the OSF at: http://doi.org/10.17605/OSF.IO/QXB4U.

## Ethics Statement

This study was carried out in accordance with the recommendations of “Institutional Review Board of the Institute of Psychology, Chinese Academy of Sciences” with written informed consent from all subjects. All subjects gave written informed consent in accordance with the Declaration of Helsinki. The protocol was approved by the “Institutional Review Board of the Institute of Psychology, Chinese Academy of Sciences.”

## Author Contributions

QL designed and organized the study. XL supervised the study. ZY and DF performed the literature search, collected the data, and prepared the manuscript. YZ and YQ revised the manuscript and provided technical support for data analysis. ZY and DF contributed equally to this work.

## Conflict of Interest Statement

The authors declare that the research was conducted in the absence of any commercial or financial relationships that could be construed as a potential conflict of interest.

## Footnotes

1 https://memebase.cheezburger.com/ragecomics
